# Improved early risk stratification of patients with ST-segment elevation myocardial infarction undergoing primary percutaneous coronary intervention using a combination of serum soluble ST2 and NT-proBNP

**DOI:** 10.1371/journal.pone.0182829

**Published:** 2017-08-10

**Authors:** Jongwook Yu, Pyung Chun Oh, Minsu Kim, Jeonggeun Moon, Yae Min Park, Kyounghoon Lee, Soon Yong Suh, Seung Hwan Han, Kyunghee Byun, Taehoon Ahn, Woong Chol Kang

**Affiliations:** 1 Cardiology, Gachon University Gil Medical Center, Incheon, Republic of Korea; 2 Gachon Cardiovascular Research Institute, Gachon University, Incheon, Republic of Korea; 3 Department of Anatomy and Cell Biology, Graduate School of Medicine, Gachon University, Incheon, Republic of Korea; 4 Functional Cellular Networks Laboratory, Lee Gil Ya Cancer and Diabetes Institute, Gachon University, Incheon, Republic of Korea; Osaka University Graduate School of Medicine, JAPAN

## Abstract

**Background:**

Although soluble suppression of tumorigenicity 2 (sST2) in serum is known to be associated with ischemic heart disease and heart failure, data regarding its prognostic impact in ST-segment elevation myocardial infarction (STEMI) is limited. We evaluated the prognostic impacts of serum sST2 and other serum biomarkers in STEMI patients undergoing primary percutaneous coronary intervention (PCI).

**Methods:**

Consecutive all 323 patients with STEMI that underwent primary PCI were enrolled. Blood tests and samples were obtained in an emergency room. The primary endpoint was 1-year major adverse cardiovascular and cerebrovascular events (MACCEs), defined as a composite of cardiovascular death, non-fatal MI, non-fatal stroke, and ischemia-driven revascularization.

**Results:**

Mean age was 59.1±13.1 years (men 84%). MACCE (20 cardiovascular deaths, 7 non-fatal MI, 4 non-fatal stroke, 7 ischemia-driven revascularizations) occurred in 38 patients (12%). After adjusting for confounding factors, Cox regression analysis revealed that high serum sST2 (>75.8 ng/mL mean value, adjusted hazard ratio 2.098, 95% CI 1.008–4.367, p = 0.048) and high serum NT-proBNP level (>400 pg/mL, adjusted hazard ratio 2.606, 95% CI 1.086–6.257, p = 0.032) at the time of presentation independently predicted MACCE within a year of primary PCI. Furthermore, when high serum sST2 level was combined with high serum NT-proBNP level, the hazard ratio of MACCE was highest (adjusted hazard ratio 7.93, 95% CI 2.97–20.38, p<0.001).

**Conclusion:**

Elevated serum levels of sST2 or NT-proBNP at the time of presentation were found to predict 1-year MACCE independently and elevated serum levels of sST2 plus NT-proBNP were associated with even poorer prognosis in patients with STEMI undergoing primary PCI.

## Introduction

ST-segment elevation myocardial infarction (STEMI) is associated with significant morbidity and mortality. Early diagnosis and proper management, especially at time of presentation in emergency room (ER), are essential for long-term prognosis. However, even after timely management, including primary percutaneous coronary intervention (PCI), patients with STEMI are still at increased risk of major adverse cardiovascular and cerebrovascular events (MACCE) [1, 2]. Therefore, early risk stratification at the time of presentation in ER is of considerable clinical importance.

Many studies have been conducted to identify more powerful predictors of the short- and long-term prognoses of STEMI, and as a result, cardiac biomarkers, such as, troponins, creatinine kinase-myocardial band (CK-MB), and natriuretic peptides are widely used to diagnose and predict prognosis in patients with STEMI [3–5].

Soluble suppression of tumorigenicity 2 (sST2), a member of the interleukin-1 receptor family, is known to be associated with ischemic heart disease and congestive heart failure (CHF). Several studies have reported sST2 is closely related with diagnosis and prognosis in patients with acute coronary syndrome, including non-STEMI [6–8]. However, data regarding the usefulness of sST2 as a prognostic marker in patients with STEMI is limited and results obtained to date conflict [9–12]. Therefore, we evaluated the prognostic impact of serum sST2 and the impacts of other biomarkers, including serum N-terminal pro-B-type natriuretic peptide (NT-proBNP), at presentation in STEMI patients undergoing primary PCI.

## Methods

### Study design and patient selection

This trial was conducted as a single center, prospective, observational study. STEMI patients aged 20 years or older treated by primary PCI from January 2011 to January 2015 were included. STEMI was diagnosed based on clinical findings, such as chest pain, 12-lead electrocardiography findings, and cardiac markers in ER setting. A well-trained study coordinator collected data using a standard protocol. The Institutional Review Board of Gachon University Gil Medical Center approved this study, which also complied with the Declaration of Helsinki (6th revision). All patients provided written informed consent prior to enrollment. Key exclusion criteria were as follows: thrombolytic therapy (n = 6), myocardial infarction (MI) caused by stent thrombosis (n = 12), history of allergy or anaphylaxis to antiplatelet agents, steel, or heparin. Patients with a history of coronary artery disease, cardiomyopathy, valvulopathy (≥ moderate), CHF or pericardial disease were also excluded (n = 84). After applying the above-mentioned inclusion and exclusion criteria, a total of 323 patients (84.0% male, mean age 59.1±13.1 years) of the originally considered 425 patients were included in the present study.

### Primary PCI

All procedures were performed according to current standard guidelines. Before PCI, patients were pre-medicated with aspirin (at least 100 mg), and a loading dose of P2Y12 receptor antagonist. Heparin was administered throughout PCI to maintain an activated clotting time of ≥250 seconds. A glycoprotein IIb/IIIa receptor blocker was administered at the discretion of the operator. Coronary angiography was performed using standard techniques. Decisions to use thrombectomy devices, intravascular ultrasound, an intra-aortic balloon pump, or percutaneous cardiopulmonary support were made by the operator. Second generation drug eluting stents were implanted in all patients. Primary success was defined as no in-hospital death, no emergency bypass surgery, and the achievement of Thrombolysis in Myocardial Infarction (TIMI) flow grade 3 in the distal portion of the infarct-related artery and the presence of <30% residual stenosis.

### Blood tests and sampling in the ER

Most blood tests were performed and recorded in accord with routine ER care of STEMI patients. Blood for two biomarkers, that is, sST2 and copeptin, was sampled in the ER and stored at -70°C. Laboratory determinations were performed by investigators unaware of patients’ clinical characteristics or outcomes. To assess sST2 and copeptin levels in serum, all ELISA reagents, working standards, and samples were prepared according to the manufacturer’s instructions (R&D Systems, Minneapolis, MN, USA). Samples were added to wells of antibody precoated microplates and were incubated adding conjugate. Substrate TMB (3,3',5,5'-tetramethylbenzidine) solution was then added, incubated for 5–10 minutes, and then an equal volume of stop solution (2N H_2_SO_4_) was added. Optical densities were measured at 450 nm. For the sST2 assay, mean intra-assay and inter-assay coefficients of variance were 1.5% and 3.2%, respectively. Serum NT-proBNP was measured using an electrochemiluminescence sandwich immunoassay and an Elecsys 2010 analyzer (Roche Diagnostics, Mannheim, Germany); coefficients of variation were 1.2% for intra-assay and 3.0% for inter-assay. Serum troponin I was measured using the ADVIA Centaur System (Bayer Diagnostics, Tarrytown, New York, USA).

### Follow up

After primary PCI, all patients were monitored in a coronary care unit for at least 24 hours. Two-dimensional transthoracic echocardiography was performed within 12 hour of the index procedure. Standard medical management including dual antiplatelet agents, beta blockers, statins, nitrates, angiotensinogen converting enzyme inhibitors or angiotensin II receptor blockers was provided by responsible physicians according to the up-to-date guidelines. After discharge, patients were followed up regularly at outpatient clinics. To assess outcomes, all medical records were reviewed and in cases lost to follow-up, families of patients were contacted by telephone.

### Study endpoint

The primary endpoint was 1-year MACCE, defined as a composite of cardiovascular death, non-fatal MI, non-fatal stroke, and ischemia-driven revascularization.

### Statistical analysis

Continuous variables are presented as means ± standard deviations for the normally distributed data or as medians (interquartile ranges) for skewed data and the significances of intergroup differences were determined using the Student’s t-test or Mann-Whitney U test. Normality of variables was evaluated using Shapiro-Wilk test (p>0.05 as normal). Categorical variables are expressed as absolute numbers and percentages, and were analyzed using the Chi-square test or Fisher’s exact test. Cox multiple regression analysis was performed to quantify relationships between MACCE and potential risk factors. The Kaplan–Meier method was used to determine cumulative MACCE-free survival rates. The analysis was performed using a computer-based statistical software package (Statistical Package for Social Sciences [SPSS^®^], Ver. 18.0 for Windows^®^; SPSS, Chicago, IL, USA). Two-sided p-values of <0.05 were considered statistically significant.

## Results

### Baseline characteristics and intergroup comparisons

Patient baseline characteristics determined in the ER and intergroup comparisons between the MACCE (+) and MACCE (−) groups are summarized in Table 1. Mean study subject age was 59.1±13.1 years and 84% were male. Of the 323 study subjects, 38 (12%) experienced MACCE. These 38 patients were significantly older and had a lower left ventricular ejection fraction (LVEF) than the 285 patients in the MACCE (-) group. However, risk factor profiles and MI severities (e.g., Killip class), or the presence of anterior MI were not significantly different in the two groups. The prevalence of diabetes mellitus tended to be higher in the MACCE (+) group.

**Table 1:** Demographic characteristics at the time of presentation in the two groups.

	Total (n = 323)	MACCE (+) (n = 38)	MACCE (-) (n = 285)	p-value
Age, years	59.1±13.1	63.7±14.4	58.5± 12.8	0.020
Male, n (%)	272 (84.0)	29 (76.3)	243 (85.3)	0.155
BMI, kg/m^2^	24.4±3.5	23.2±4.0	24.5±3.4	0.039
Systolic BP, mmHg	127.6±29.4	120.1±41.2	128.6±27.4	0.225
Diastolic BP, mmHg	76.6±18.5	72.9±27.3	77.1±16.9	0.362
Heart rate, per min	75.9±20.0	76.4±30.5	75.9±18.2	0.912
Hypertension, n (%)	148 (46.0)	17 (44.7)	131 (46.0)	0.887
Diabetes mellitus, n (%)	73 (22.6)	13 (34.2)	60 (21.0)	0.069
Smoking, n (%)	183 (57.0)	19 (51.4)	164 (58.0)	0.460
Dyslipidemia, n (%)	36 (11.1)	4 (10.5)	32 (11.2)	0.897
Killip class, n (%)				0.444
1	261 (80.8)	32 (84.2)	229 (80.3)	
2	9 (2.8)	2 (5.3)	7 (2.5)	
3	42 (13.0)	4 (10.5)	38 (13.3)	
4	11 (3.4)	0 (0.0)	11 (3.9)	

MACCE: major adverse cardiovascular and cerebrovascular events, BMI: body mass index, BP: blood pressure

Table 2 showed laboratory findings according to presence or absence of the MACCE. The MACCE (+) group had significantly higher plasma random glucose and HbA1clevels at presentation. In the subgroup with a history of diabetes mellitus (n = 73), random plasma glucose was significantly higher in the MACCE (+) group than in the MACCE (-) group (224.0 [206.0–285.5] vs. 185.5 [149.3–243.3] mg/dL, respectively, p = 0.015), however, there was no significant difference of HbA1c between the MACCE (+) and MACCE (-) groups (7.9±1.6 vs. 7.3±1.3%, respectively, p = 0.293). The mean level of sST2 was not different between subjects with and without diabetes (75.6±26.9 vs. 76.4±31.4 ng/mL, respectively, p = 0.826). Furthermore, there were no significant correlations between the levels of sST2 and random plasma glucose or HbA1c. The levels of troponin I, CK-MB, and NT-proBNP at the time of presentation were significantly higher in the MACCE (+) group than in the MACCE (-) group. Initial sST2 level tended to be higher in the MACCE (+) group. Although there were no significant difference of serum transaminase levels between the two groups, hypoxic livery injury (serum transaminase level > twice the upper limit of normal [13]) tended to be more frequent in the MACCE (+) group.

**Table 2:** Laboratory findings at the time of presentation in the two groups.

	Total (n = 323)	MACCE (+) (n = 38)	MACCE (-) (n = 285)	p-value
Glucose, mg/dL	151.0 (127.0–195.0)	210.0 (151.5–285.5)	148.0 (125.0–184.0)	<0.001
HbA1c, %	6.4±1.5	7.5±2.3	6.27±1.2	0.022
AST, U/L	30.0 (22.0–64.3)	36.0 (24.0–102.0)	29.0 (22.0–57.0)	0.090
ALT, U/L	26.0 (18.0–45.3)	26.0 (19.0–49.0)	26.0 (18.0–44.5)	0.785
Hypoxic livery injury^†^, n (%)	74 (23.0)	13(35.1)	61 (21.4)	0.062
Total protein, g/dL	7.0±0.6	7.0±0.7	7.0±0.6	0.758
hs-CRP, mg/dL	0.17 (0.08–0.53)	0.41 (0.12–1.3)	0.16 (0.07–0.45)	0.004
sST2, ng/mL	75.8±27.8	84.0±29.7	74.7±27.6	0.052
Copeptin, pg/mL	0.59 (0.44–0.79)	0.60 (0.37–1.17)	0.59 (0.46–0.77)	0.610
Hemoglobin, g/dL	14.4±2.2	13.8±3.0	14.5±2.1	0.058
Total bilirubin, mg/dL	0.7±0.3	0.7±0.4	0.7±0.3	0.620
Creatinine, mg/dL	0.9 (0.8–1.0)	0.9 (0.8–1.1)	0.9 (0.8–1.0)	0.296
HDL-C, mg/dL	40.2±10.4	39.2±12.8	40.3±10.1	0.551
LDL-C, mg/dL	109.5±34.5	106.8±36.7	109.8±34.2	0.638
NT-proBNP, pg/mL	167 (44.9–693.7)	734.7 (221.9–2346.5)	145.6 (39.5–564.2)	<0.001
CK-MB, ng/mL	2.4 (1.2–7.6)	6.9 (1.2–26.9)	2.2 (1.1–6.5)	0.018
Troponin I, ng/mL	0.11 (0.02–0.83)	0.79 (0.07–5.33)	0.10 (0.02–0.55)	<0.001
Albumin, g/dL	4.3±0.7	4.5±1.8	4.3±0.4	0.535

MACCE: major adverse cardiovascular and cerebrovascular events, HbA1c: hemoglobin A1c, AST: aspartate transaminase, ALT: alanine transaminase, hsCRP: high sensitivity C-reactive protein, sST2: soluble suppression of tumorigenicity 2, HDL-C: high density lipoprotein-cholesterol, LDL-C: low density lipoprotein-cholesterol, NT-proBNP: N-terminal pro-B-type natriuretic peptide, CK-MB: creatinine kinase-myocardial band

^†^Hypoxic liver injury was defined as an elevation of serum transaminase level more than twice the upper limit of normal [13].

### Primary PCI findings

A summary of angiographic, procedural, and echocardiographic data is provided in Table 3. Although baseline characteristics, including extent of coronary artery disease, infarct-related artery, and baseline TIMI flow grade were similar in the two groups, final TIMI flow grade, primary success rate, and LVEF were significantly lower in the MACCE (+) group. Serum sST2 levels were not significantly different among the groups according to the number of affected coronary arteries (75.9±27.6 ng/mL for 1 vessel disease, 74.2±29.7 ng/mL for 2 vessels disease, 77.9±26.1 ng/mL for 3 vessels disease, p = 0.664). Serum sST2 levels according to IRA (infarct-related artery) were also not statistically significant. (74.8±29.0 ng/mL for left main or left anterior descending artery, 77.0±29.6 ng/mL for left circumflex or right coronary artery, p = 0.471). Symptom to presentation time was adequately reported only in 72 patients (7 with MACCE and 65 without MACCE, 262 [146–633] vs. 239 [125–609] min, respectively, p = 0.64).

**Table 3:** Primary PCI results and transthoracic echocardiographic findings.

	Total (n = 323)	MACCE (+) (n = 38)	MACCE (-) (n = 285)	p-value
Extent of CAD, n (%)				0.417
1-vessel	123 (38.0)	11 (28.9)	112 (39.3)	
2-vessel	118 (36.5)	17 (44.7)	101 (35.4)	
3-vessel	82 (25.3)	10 (26.3)	72 (25.3)	
Multivessel disease	200 (62.0)	27 (71.1)	173 (60.7)	0.217
Infarct-related artery, n (%)				0.440
Left main artery	1 (0.3)	0 (0.0)	1 (0.1)	
Left anterior descending artery	184 (56.9)	26 (68.4)	158 (55.4)	
Left circumflex artery	25 (7.7)	3 (7.8)	22 (7.7)	
Right coronary artery	113 (34.9)	9 (23.6)	104 (36.4)	
Baseline TIMI flow grade, n (%)				0.870
0–2	269 (83.2)	32 (84.2)	237 (83.1)	
3	54 (16.7)	6 (15.7)	48 (16.8)	
Final TIMI flow grade, n (%)				<0.001
0–2	28 (8.6)	11 (28.9)	17 (5.9)	
3	295 (91.3)	27 (71.0)	268 (94.0)	
Primary success, n (%)	286 (88.5)	18 (47.3)	268 (94.0)	<0.001
LVEF, %	50.1±11.9	43.3±16.8	50.9±10.9	0.015

PCI: percutaneous coronary intervention, MACCE: major adverse cardiovascular and cerebrovascular events, CAD: coronary artery disease, LVEF: left ventricular ejection fraction

### Associations between adverse outcomes and biomarkers including sST2 and NT-proBNP

During a year following index PCI, 38 MACCEs occurred (20 cardiovascular deaths, 7 non-fatal MI, 4 non-fatal stroke and 7 ischemia-driven revascularizations), an event rate of 12.0%. Cox multiple regression analysis was used to identify independent predictors for MACCE after index PCI, and results are shown in Table 4. Random plasma glucose level, or sST2 level was categorized into high and low groups according to median or mean level (random plasma glucose level, 151 mg/dL; sST2, 75.8 ng/mL) because the prognostic cut-off values had not been established. However, well-known prognostic cut-off values were used for classifying levels of NT-proBNP, troponin I and high sensitivity C-reactive protein (hsCRP) (400 pg/mL, 0.1 ng/mL, and 1 mg/dL, respectively) [14–17].

**Table 4:** Cox regression analysis findings for predictors for 1-year MACCE after primary PCI.

Variables	HR	95% CI	p-value
Univariate analysis			
Age (10-year increase)	1.36	1.06–1.74	0.016
Diabetes mellitus	1.88	0.96–3.67	0.066
Low LVEF (<40%)	3.12	1.54–6.30	0.002
Low final TIMI flow grade (<3)	5.16	2.55–10.41	<0.001
High sST2 (>75.8 ng/mL)	1.965	1.006–3.842	0.048
High NT-proBNP (>400 pg/mL)	3.736	1.902–7.399	<0.001
High troponin I (>0.1 ng/mL)	2.376	1.179–4.791	0.016
High hsCRP (>1 mg/dL)	2.448	1.224–4.895	0.011
High random plasma glucose level (>151mg/dL)	3.344	1.578–7.087	0.002
Hypoxic liver injury (serum transaminase >80 U/L)	1.946	0.991–3.821	0.053
Multivariate analysis			
Low final TIMI flow grade (<3)	2.556	1.049–6.228	0.039
High sST2 (>75.8 ng/mL)	2.098	1.008–4.367	0.048
High NT-proBNP (>400 pg/mL)	2.606	1.086–6.257	0.032
High random plasma glucose level (>151mg/dL)	3.737	1.586–8.806	0.003

MACCE: major adverse cardiovascular and cerebrovascular events, PCI: percutaneous coronary intervention, HR: hazard ratio, LVEF: left ventricular ejection fraction, sST2: soluble suppression of tumorigenicity 2, NT-proBNP: N-terminal pro-B-type natriuretic peptide, hsCRP: high sensitivity C-reactive protein

**Fig 1:**
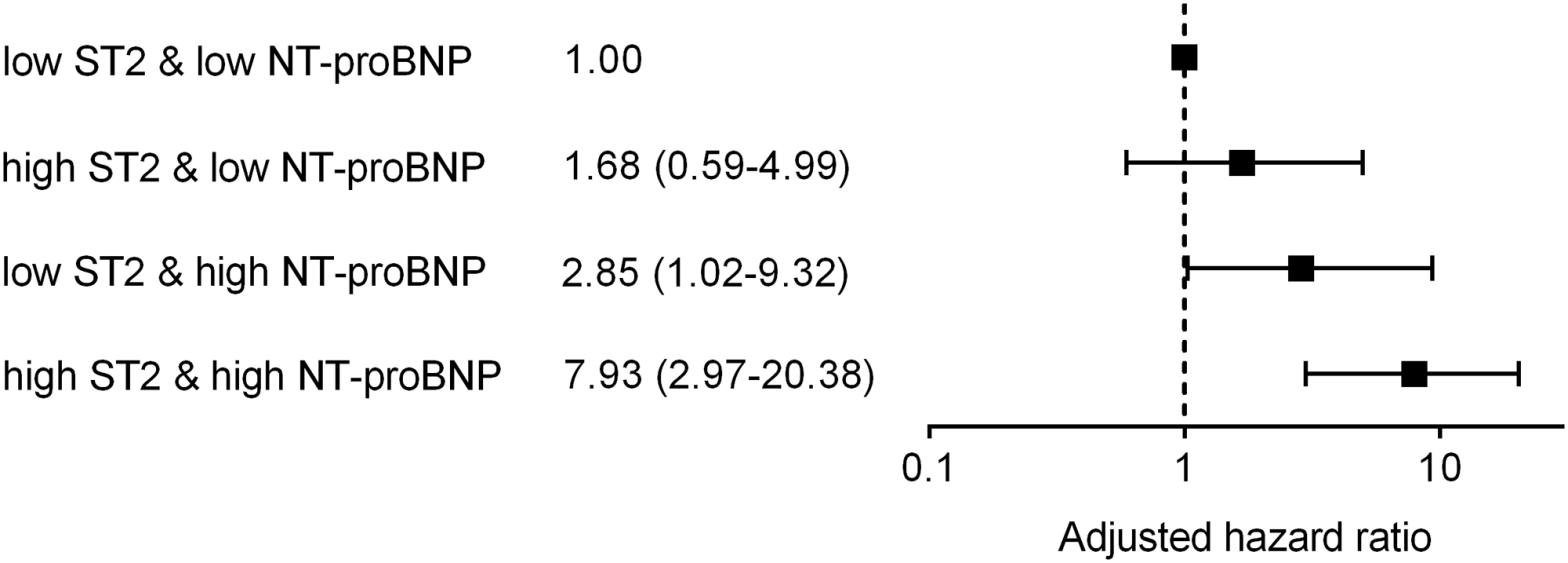
High sST2 and high NT-proBNP levels in combination had the highest adjusted hazard ratio.

**Fig 2:**
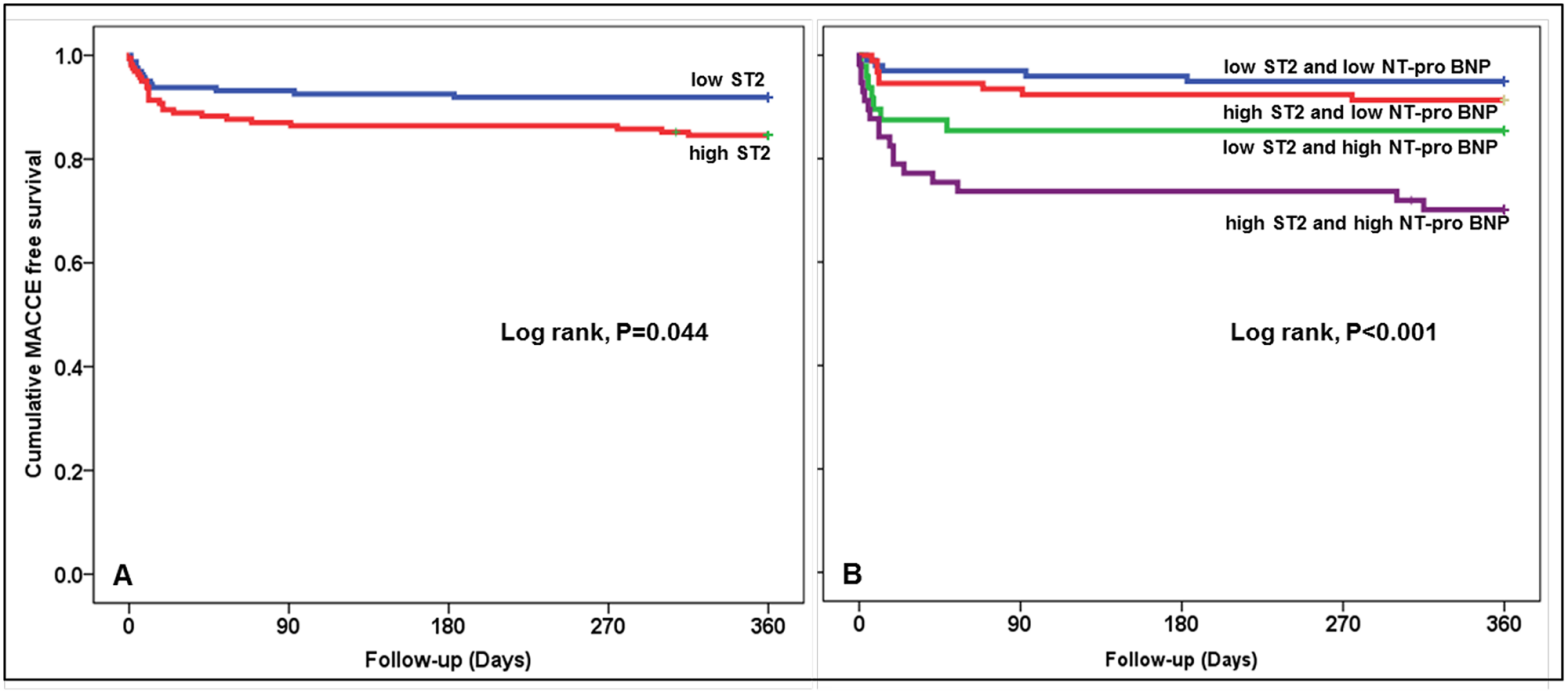
Kaplan-Meier survival curves for MACCE during a year following primary PCI showed high sST2 level was associated with a poorer prognosis (A) and that high sST2 and high NT-proBNP levels in combination were associated with a worst prognosis than any other levels of combination (B).

After adjusting for age, diabetes mellitus, final TIMI flow grade, hypoxic liver injury, hsCRP level, and troponin I level, high serum sST2 level (>75.8 ng/mL, adjusted hazard ratio2.098, 95% CI1.008–4.367, p = 0.048) and high serum NT-proBNP level (>400 pg/mL, adjusted hazard ratio 2.606, 95% CI1.086–6.257, p = 0.032) at presentation were found to independently predict 1-year MACCE. Of interest, high sST2 combined with high NT-proBNP had the highest adjusted hazard ratio (7.934, 95% CI 2.974–20.381, p<0.001) (Fig 1). Notably, serum troponin I level, hsCRP level and copeptin level at presentation failed to predict MACCE. The survival probability plots of 1-year MACCE revealed high serum sST2 was associated with poorer prognosis than low sST2 and combined high sST2 and NT-proBNP was associated with even poorer prognosis than any other combinations (Fig 2).

## Discussion

### Principal findings

The present study was undertaken to evaluate the prognostic impacts of serum sST2 and other serum biomarkers in STEMI patients undergoing primary PCI. Its main findings are as follows. (1) High serum sST2 (>75.8 ng/mL mean value) and NT-proBNP (>400 pg/mL) levels obtained at the time of presentation in the ER independently predicted MACCE during a year following primary PCI. (2) Traditional cardiac markers, such as CK-MB and troponin I level, determined at the time of presentation, failed to predict MACCE. (3) Serum sST2 combined with serum NT-proBNP best predicted MACCE. (4) Survival probability plots of MACCE during the first year after PCI revealed that combined high serum sST2 and NT-proBNP was associated with the worst prognosis. Our results indicate high serum sST2 and NT-proBNP levels at the time of presentation in the ER are highly associated with poor prognosis and that they are probably better early prognostic markers than other parameters, including serum CK-MB, troponin I, and copeptin levels in patients with STEMI treated by primary PCI.

### Prognostic biomarkers for patients with STEMI

Despite marked improvements in outcomes over the past few decades, relapse rates of adverse events are still unacceptably high for patients with STEMI [1, 2], and thus, it is of considerable important efforts be made to continue to develop tools that identify patients at high risk of adverse outcomes who might benefit more from enhanced monitoring or treatment. Moreover, early recognition of these patients at the time of presentation in the ER is crucial to reduce adverse outcomes. Many studies have been conducted to identify prognostic markers in patients with STEMI [6, 10, 11]. One study that included up to 1258 patients with acute MI showed that several candidate markers, that is, NT-proBNP, midregional proatrial natriuretic peptide (MR-proANP), sST2, troponin T, myeloperoxidase (MPO), hsCRP, and pregnancy-associated plasma protein-A (PAPP-A), were significantly associated with 30-day risk of cardiovascular death or CHF after adjusting for clinical predictors [18].

### Prognostic value of serum sST2

sST2 was evaluated by Weinberg EO et al. in 2002 [19] and has widely been studied in diabetes mellitus and cardiovascular disease. This notable cardiac biomarker is being increasingly used in patient care, but its clinical and pathophysiologic implications have not been thoroughly determined. Recently, Lin et al demonstrated diabetic patients had significantly higher levels of sST2 compared with normal subjects and elevated sST2 levels had increased risk of having diabetes. However, in the present study, sST2 levels were not different in patients with or without diabetes and could not reflect glycemic status, suggesting sST2 might have different roles in glucose metabolism and the setting of myocardial injury. Furthermore, sST2 is being actively investigated in the contexts of the prognosis and diagnosis of CHF [20–23], and relatively little information is available about its role in patients with STEMI. Shimpo M et al. measured serum sST2 levels at presentation in 810 patients with STEMI [9]. It was found sST2 levels were higher in patients that succumbed or experienced CHF before 30 days had elapsed after PCI, and multivariate analysis showed sST2 remained predictive of mortality at 30 days after adjusting for age, heart rate, blood pressure, location of infarction, Killip class, and time from symptom onset. However, this independent association disappeared after adjusting for BNP and troponin I. This finding is inconsistent with our findings that high sST2 level at the time of presentation independently predicted MACCE after adjusting for confounding markers. On the other hand, in another cohort in the same study, additional samples were taken at later times and in this cohort serum sST2 at 12 hours after presentation independently predicted mortality. Accordingly, the investigators concluded that sST2 checked at 12 hours after presentation, was associated with mortality after STEMI independently of established clinical indicators. Another point of difference between this previous study and the present study is that the risks of marker combinations, such as, sST2 coupled with troponin I or BNP, were not assessed.

The CLARITY-TIMI 28 trial [11] revealed sST2 and NT-proBNP were found to be complementary in STEMI. In this trial, patients with STEMI were enrolled regardless of primary PCI and it was found serial changes of blood levels of sST2 and NT-proBNP after MI differed. Blood samples were drawn at presentation and several days after MI during angiography, and whereas sST2 modestly decreased between samplings, mean NT-proBNP increased by several folds. In the same study, angiographically determined low TIMI flow grade and TIMI myocardial perfusion grade were found to be more associated with higher sST2 levels than NT-proBNP levels, whereas NT-proBNP levels were more closely associated with LVEF. These findings suggest serum **s**ST2 level reflects the amount of injured tissue and is associated necrosis and inflammatory events, whereas NT-proBNP is associated cardiac mechanical stress. Accordingly, this previously published data indicated these two markers might act in a complementary manner to predict adverse events in STEMI. Mean serum concentrations of sST2 and NT-proBNP in patients that later experienced cardiovascular death or CHF were higher than those of patients that did not experience these outcomes [11], and similarly, these marker levels were higher in patients that experienced stroke than in those that did not [24]. Thus, it would appear that high sST2 and NT-proBNP in combination might be reasonably expected to predict MACCE after STEMI.

Interestingly, although serum copeptin is a well-known cardiac marker with a high negative predictive value in patients with STEMI [18, 25, 26], we found initial serum copeptin measured in the ER setting did not predict long-term prognosis in STEMI patients.

### Limitations

Several limitations of the present study should be considered. First, this single center, observational study was conducted using a relatively small Korean cohort. Nevertheless, the cohort was homogeneous and all study subjects were STEMI patients that underwent primary PCI and were managed using the same protocol. Second, all blood tests and samplings were performed at the time of ER presentation, and thus, no subsequent blood tests were performed for sST2 or copeptin, because our primary aim was to identify early prognostic markers for STEMI in the ER setting. However, this prevented investigating associations between serial changes in sST2 and copeptin levels and clinical outcomes. Third, the time from the first symptoms of MI to presentation in the hospital or PCI would be important, because it might affect sST2 or NT-proBNP levels and has a considerable impact on clinical outcomes. Unfortunately, the time interval was reported only in a portion of subjects and thus its prognostic implication could not be determined in this study. Finally, the cut-off values for sST2 suggested by the present study cannot be compared directly with those of previous studies, because sST2 measurement methods differed.

## Conclusion

In conclusion, high sST2 and NT-proBNP levels obtained at the time of presentation in the ER were found to independently predict MACCE during a year following PCI, and combined high sST2 and NT-proBNP was associated with the worst prognosis.
